# Technical Note: Monte Carlo calculations of the AAPM TG‐43 brachytherapy dosimetry parameters for a new titanium‐encapsulated Yb‐169 source

**DOI:** 10.1002/acm2.12111

**Published:** 2017-06-06

**Authors:** Francisco J. Reynoso, John J. Munro III, Sang Hyun Cho

**Affiliations:** ^1^ Department of Radiation Physics The University of Texas MD Anderson Cancer Center Houston TX USA; ^2^ Source Production & Equipment Co. St. Rose LA USA; ^3^ Department of Imaging Physics The University of Texas MD Anderson Cancer Center Houston TX USA; ^4^ Present address: Department of Radiation Oncology Washington University School of Medicine St. Louis MO USA; ^5^ Present address: Montrose Technology Inc. North Andover MA USA

**Keywords:** gold nanoparticle‐aided radiation therapy, Monte Carlo method, TG‐43, Yb‐169 source

## Abstract

Due to a number of distinct advantages resulting from the relatively low energy gamma ray spectrum of Yb‐169, various designs of Yb‐169 sources have been developed over the years for brachytherapy applications. Lately, Yb‐169 has also been suggested as an effective and practical radioisotope option for a novel radiation treatment approach often known as gold nanoparticle‐aided radiation therapy (GNRT). In a recently published study, the current investigators used the Monte Carlo N‐Particle Version 5 (MCNP5) code to design a novel titanium‐encapsulated Yb‐169 source optimized for GNRT applications. In this study, the original MC source model was modified to accurately match the specifications of the manufactured Yb‐169 source. The modified MC model was then used to obtain a complete set of the AAPM TG‐43 parameters for the new titanium‐encapsulated Yb‐169 source. The MC‐calculated dose rate constant for this titanium‐encapsulated Yb‐169 source was 1.19 ± 0.03 $cGy\cdot h^{-1}\cdot U^{-1}$, indicating no significant change from the values reported for stainless steel‐encapsulated Yb‐169 sources. The source anisotropy and radial dose function for the new source were also found similar to those reported for the stainless steel‐encapsulated Yb‐169 sources. The current results suggest that the use of titanium, instead of stainless steel, to encapsulate the Yb‐169 core would not lead to any major change in the dosimetric characteristics of the Yb‐169 source. The results also show that the titanium encapsulation of the Yb‐169 source could be accomplished while meeting the design goals as described in the current investigators’ published MC optimization study for GNRT applications.

## 1 INTRODUCTION

Over the years, various designs of Yb‐169 sources have been described in the published literature.^1^, ^2^, ^3^, ^4^, ^5^, ^6^, ^7^, ^8^, ^9^, ^10^, ^11^, ^12^, ^13^ As summarized previously,^13^ the relatively low energy photon spectrum of Yb‐169 would provide multiple advantages including the possibility of *in vivo* shielding of essential organs and tissues via shielded applicator (e.g., using 0.5–1.0 mm thick lead foils in the applicator system to reduce bladder and rectal doses in gynecological malignancies^3^), reduced radiation exposure to personnel, simplified high dose rate (HDR) room shielding, streamlined after‐loading units, and overall reduced costs.^1^, ^2^, ^3^, ^4^, ^7^, ^9^, ^10^, ^11^, ^12^ Additionally, Yb‐169 has been suggested as an almost ideal radioisotope for the brachytherapy implementation of so‐called gold nanoparticle‐aided radiation therapy (GNRT),^13^, ^14^ because its gamma ray spectrum (average energy of 93 keV just above the K‐absorption edge of gold) can lead to more advantageous (e.g., larger or/and more uniform) dose enhancement characteristics with gold nanoparticles (GNPs) than other radioisotopes being used for brachytherapy purposes (e.g., Ir‐192, I‐125, Pd‐103, etc.). To follow‐up on this suggestion, we designed a new titanium‐encapsulated Yb‐169 source optimized for GNRT applications,^13^ based on our Monte Carlo (MC) investigation of the effects of the Yb‐169 source encapsulation on the photon spectra, and more importantly the secondary electron spectra that are directly responsible for the dose enhancement characteristics for a given concentration of GNPs. After our initial MC source design study,^13^ we proceeded to produce novel titanium‐encapsulated Yb‐169 sources in collaboration with a source manufacturer (Source Production & Equipment Co., Inc., St. Rose, LA, USA).

In the current MC study, we determined a complete set of brachytherapy dosimetry parameters for the aforementioned titanium‐encapsulated Yb‐169 source model, following the American Association of Physicists in Medicine (AAPM) Task Group 43 (TG‐43) formalism.^15^, ^16^ The key results from the current investigation were compared with those from the previous investigations of various Yb‐169 source models, in light of GNRT as well as general brachytherapy applications.

## 2 METHODS

### 2.A Source design

**Figure 1:**
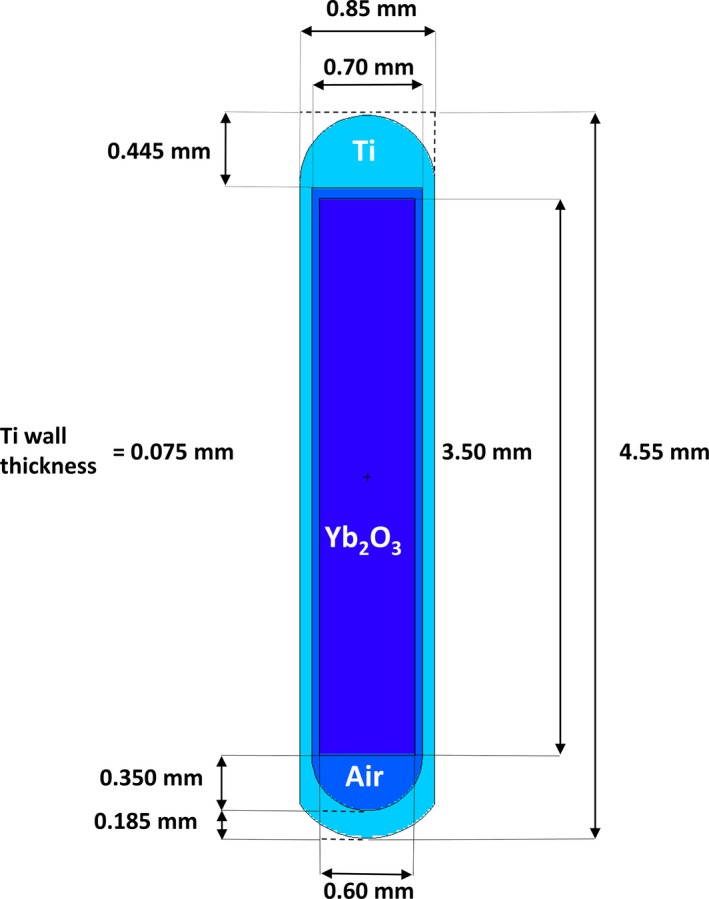
MCNP5 model of the manufactured titanium‐encapsulated ^169^Yb source per the detailed specifications from the source manufacturer (Source Production & Equipment Co., Inc., St. Rose, LA). As shown, an active Ytterbium core is 3.5 mm in length and 0.6 mm in diameter. For the anisotropy data, *θ* = 0 is corresponding to the vertical axis of the source in the negative (downward) direction in the diagram. Figure is drawn to scale.

As described in our previous publication,^13^ the new Yb‐169 source optimized for GNRT applications was designed similar to a previously investigated HDR Yb‐169 source,^7^, ^12^, ^17^ with the exception of the encapsulation material (i.e., titanium vs. stainless steel). While its specific design was slightly different from that described in our previous publication,^13^ the new Yb‐169 source manufactured from this investigation maintained the key features of our original source design, most notably the titanium encapsulation as compared to a more conventional stainless steel encapsulation. As depicted in Fig. 1, the new Yb‐169 seed source had an active Ytterbium core (3.5 mm in length, 0.6 mm in diameter, and 7.0 mg mm^−3^ in density) encapsulated by American Society for Testing and Materials (ASTM) grade 2 titanium (4.54 mg mm^−3^ in density). This source had an air gap between the active Ytterbium core and titanium encapsulation, which was included in our MC model (Fig. 1) following the specifications provided by the source manufacturer. It should be noted that, while intended for eventual HDR applications, Yb‐169 sources produced during the current investigation had their activities on the order of 10 mCi for the ease of handling and testing.

### 2.B Monte Carlo calculations of TG‐43 parameters

The MC radiation transport code, Monte Carlo N‐Particle Version 5 (MCNP5), was used to compute all the necessary quantities to characterize the Yb‐169 source as defined by TG‐43. The source and encapsulation geometry were modeled exactly as shown in Fig. 1. The active region of the source was modeled with a uniform activity distribution. Table 1 shows the Yb‐169 photon spectrum used for the current MC study excluding all photons with intensity lower than 0.1% and energy lower than 5 keV as specified in TG‐43.^15^, ^16^ Two different MCNP models were developed to compute all TG‐43 parameters: *S*_*K*_ the air‐kerma strength of the source (*μ*Gy·m^2^/h), Λ the dose‐rate constant in water (1/m^2^), $G\left(r,\theta \right)$ the geometry function (1/m^2^), $g_{L}\left(r\right)$ the radial dose function, $F\left(r,\theta \right)$ the anisotropy function.

**Table 1:** Yb‐169 photon spectrum including all photons with yields greater than 0.1% and ignoring all dosimetrically irrelevant gamma rays below 5 keV

Energy (*KeV*)	Photons per disintegration
49.77	0.532
50.74	0.94
57.30	0.0993
57.51	0.192
57.90	0.00379
59.03	0.0647
59.21	0.0172
63.12	0.442
93.62	0.0261
109.78	0.1747
118.19	0.01869
130.52	0.1131
177.21	0.2216
197.96	0.358
261.08	0.01715
307.74	0.1005
TOTAL	3.32083

The air‐kerma strength *S*_*k*_ was calculated with the Yb‐169 source centered in a 130 cm radius spherical phantom *in vacuo*. The air‐kerma rate was determined at the reference point *θ*_*o*_ *=* 90^*o*^ at a distance *d = 100 cm* using the MCNP5 energy deposition F6 tally with units of MeVg^−1^photon^−1^. This region was defined by first delineating the region at *d = 100 cm* from the center of the source by defining two concentric spheres with radii of 97.5 cm and 102.5 cm. The angular constraint of *θ*_*o*_ *=* 90^*o*^ was defined by using two cones with vertex angles of *θ =* 88^*o*^ one aligned with the +z‐axis and the other aligned with the –z‐axis. This defined a *5* cm ring tally centered at *d = 100 cm* with *θ*_*o*_ *=* 90^*o*^ *±* 2^*o*^. The MCNP output was the air‐kerma per source photon $\left(K_{MC}\right)$ in units of *MeV · g* ^*−1*^ *· photon* ^*−1*^. The air‐kerma rate was then calculated from *K*_*MC*_ and converted to units of *cGy · mCi* ^*−1*^ *· h* ^*−1*^ by:

(1) $$\dot{K}\left(d,\theta \right)=K_{MC}\cdot I_{\gamma }\cdot 2.134\times 10^{3}\frac{cGy}{mCi\cdot h}$$

where *I*_*γ*_ is the total number of photons per disintegration of the source. The air‐kerma rate may also be written in terms of the unit U (*cGy · cm* ^*2*^ *· h* ^*−1*^) as specified in TG‐43.^15^, ^16^

The dose distribution surrounding the source was computed by simulating the source centered in a spherical water phantom with a radius of 50 cm, an appropriate size to approximate full‐scatter conditions of a semi‐infinite water phantom. An array of tally regions was modeled to collect the dose at radial distances of 0.5 cm and 1–10 cm in 1 cm steps and at angles between *θ* = 0^o^ and *θ* = 180^o^ in 10^o^ steps. This was accomplished by generating spherical shells with mean radii at the desired radial distance (i.e., 0.5 cm and 1–10 cm in 1 cm steps). The thickness of each shell was calculated to be as thin as possible to appropriately approximate the detection region while maximizing collection efficiency during the MC simulation. The criteria were developed by Luxton et al.^18^ and compare the factor $R_{V}={\left(R_{1}^{3}+R_{2}^{3}/2\right)}^{\frac{1}{3}}$ that subdivides each shell bounded by the inner radius *R*_1_ and outer radius *R*_2_ into smaller shells of equal volume, with the mean radius of the shell *R*_*M*_ = (*R*_1_ + *R*_2_)/2. The calculated dose for a shell approximates the dose at that mean radius of the shell only if the two factors differ by less than 1%, i.e., $\left|{\left(R_{V}/R_{M}\right)}^{2}-1\right|<0.01$.

The angular dependence of each tally region was defined by using concentric cones about the +z‐axis and –z‐axis to restrict collection along the desired angle from *θ* = 0^o^ to *θ* = 180^o^ in 10^o^ steps. For the *θ* = 0^0^ region, a cone along the –z‐axis with vertex angle of 2^o^ defined the *θ* = 0^o^ region; a similar cone along the +z‐axis defined *θ* = 180^o^. The regions between 10^o^ and 170^o^ are defined by concentric cones centered at the desired angle with an angular opening ± 4^o^, e.g., for the *θ* = 10^o^ two concentric cones centered along the –z‐axis with vertex angles of 8^o^ and 12^o^ defined the desired tally region. The same method was used to define all of the angles and using concentric cones with the appropriate vertex angles to define each region in 10^o^ (i.e., $\left(8^{o},12^{o},18^{o},22^{o},28^{o},32^{o},\ldots \right)$. The result is a series of ring tallies that collect the dose distribution surrounding the source.

## 3 RESULTS

MCNP5 calculations resulted in the air‐kerma strength as $S_{K}=1.15\pm 0.03U\cdot mCi^{-1}$ and the dose rate at the reference point $\dot{D}\left(r_{o},\theta_{o}\right)$as $1.37\pm 0.02cGy\cdot mCi^{-1}\cdot h^{-1}$. Accordingly, the dose‐rate constant was calculated as $\Lambda =1.19\pm 0.03cGy\cdot h^{-1}\cdot U^{-1}$. This value can be compared to other reported values for Yb‐169 source models: Λ = 1.210 ± 0.050,^11^ Λ = 1.204 ± 0.004,^19^ Λ = 1.19 ± 0.03,^7^ Λ = 1.12 ± 0.04,^12^ Λ = 1.170 ± 0.010, and Λ = 1.191 ± 0.007 *cGy · h* ^*−1*^ *· U* ^*−1*^.^20^

**Figure 2:**
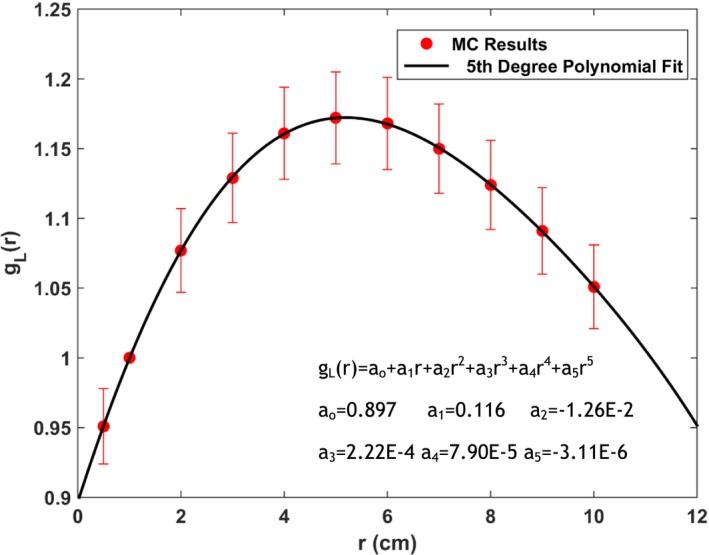
Calculated radial dose function for the current Yb‐169 source and 5^th^ degree polynomial fit along with corresponding fitting parameters.

**Figure 3:**
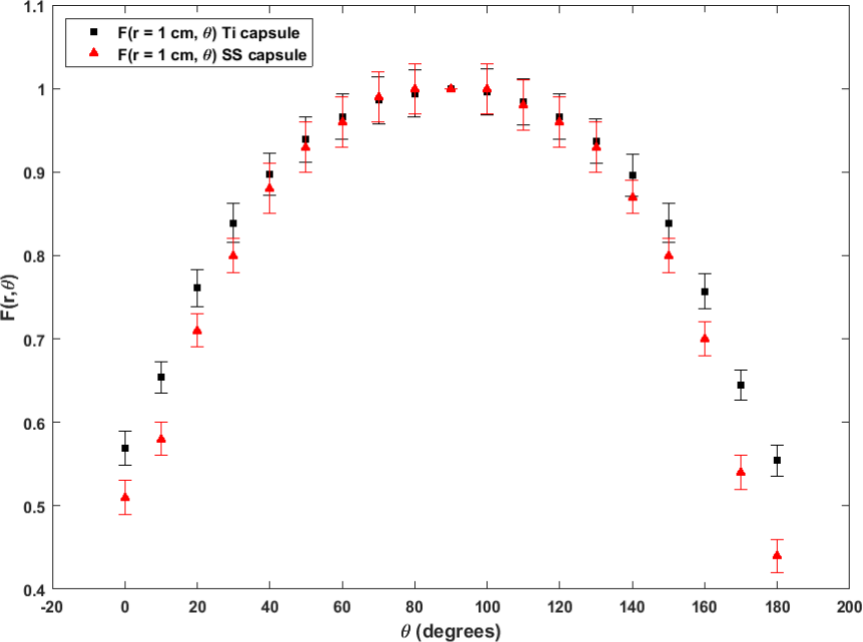
Comparison of the source anisotropy data at r = 1.0 cm, $F\left(r=1.0\;cm,\theta \right)$, between the current titanium encapsulated Yb‐169 source and a previously reported stainless steel encapsulated Yb‐169 source.^7^

The geometry function $G_{L}\left(r,\theta \right)$ (Table 2) represents the effective inverse‐square correction based on the line‐source approximation. The function shows it effectively becomes point sources for r≥5cm. The radial dose functions are shown in Table 3 and the fit to 5^th^ order polynomial as specified in the updated report of TG‐43^15^ is shown in Fig. 2. Table 4 presents the values of 2D anisotropy function $F\left(r,\theta \right)$ from the current study. Figure 3 shows a comparison of the source anisotropy at r = 1.0 cm between the current titanium‐encapsulated Yb‐169 source and a previously described stainless steel encapsulated Yb‐169 source.^7^

**Table 2:** Calculated geometry function $G_{L}\left(r,\theta \right)$. The length of an active ytterbium core was taken as 3.5 mm for the line‐source approximation

Polar angle θ(deg.)	*r* (cm)										
	0.5	1	2	3	4	5	6	7	8	9	10
0	4.558	1.032	0.252	0.111	0.063	0.040	0.028	0.020	0.016	0.012	0.010
10	4.530	1.030	0.252	0.111	0.063	0.040	0.028	0.020	0.016	0.012	0.010
20	4.450	1.026	0.252	0.111	0.063	0.040	0.028	0.020	0.016	0.012	0.010
30	4.337	1.021	0.251	0.111	0.063	0.040	0.028	0.020	0.016	0.012	0.010
40	4.212	1.014	0.251	0.111	0.063	0.040	0.028	0.020	0.016	0.012	0.010
50	4.092	1.006	0.250	0.111	0.063	0.040	0.028	0.020	0.016	0.012	0.010
60	3.989	1.000	0.250	0.111	0.062	0.040	0.028	0.020	0.016	0.012	0.010
70	3.912	0.995	0.250	0.111	0.062	0.040	0.028	0.020	0.016	0.012	0.010
80	3.864	0.991	0.249	0.111	0.062	0.040	0.028	0.020	0.016	0.012	0.010
90	3.848	0.990	0.249	0.111	0.062	0.040	0.028	0.020	0.016	0.012	0.010

**Table 3:** Radial dose function $g_{L}\left(r,\theta \right)$ values

*r* (cm)	*g_L_ (r)* for Ti design	
0.5	0.951	± 0.027
1	1.000	
2	1.077	± 0.030
3	1.129	± 0.032
4	1.161	± 0.033
5	1.172	± 0.033
6	1.168	± 0.033
7	1.150	± 0.032
8	1.124	± 0.032
9	1.091	± 0.031
10	1.051	± 0.030

**Table 4:** 2D Anisotropy function $F\left(r,\theta \right)$ values

Polar angle θ (degrees)	*r* (cm)																					
	0.5		1		2		3		4		5		6		7		8		9		10	
0	0.575	± 0.020	0.569	± 0.020	0.613	± 0.020	0.659	± 0.021	0.703	± 0.022	0.725	± 0.022	0.752	± 0.023	0.763	± 0.023	0.770	± 0.023	0.817	± 0.025	0.814	± 0.025
10	0.637	± 0.018	0.654	± 0.019	0.702	± 0.020	0.740	± 0.021	0.766	± 0.022	0.787	± 0.022	0.805	± 0.023	0.820	± 0.023	0.831	± 0.024	0.842	± 0.024	0.847	± 0.024
20	0.750	± 0.021	0.761	± 0.022	0.792	± 0.022	0.816	± 0.023	0.833	± 0.024	0.846	± 0.024	0.857	± 0.024	0.867	± 0.025	0.874	± 0.025	0.880	± 0.025	0.886	± 0.025
30	0.836	± 0.024	0.839	± 0.024	0.859	± 0.024	0.875	± 0.025	0.885	± 0.025	0.892	± 0.025	0.901	± 0.025	0.907	± 0.026	0.912	± 0.026	0.915	± 0.026	0.918	± 0.026
40	0.896	± 0.025	0.897	± 0.025	0.910	± 0.026	0.918	± 0.026	0.924	± 0.026	0.930	± 0.026	0.934	± 0.026	0.938	± 0.027	0.940	± 0.027	0.943	± 0.027	0.945	± 0.027
50	0.938	± 0.027	0.939	± 0.027	0.945	± 0.027	0.951	± 0.027	0.954	± 0.027	0.957	± 0.027	0.959	± 0.027	0.962	± 0.027	0.963	± 0.027	0.964	± 0.027	0.966	± 0.027
60	0.968	± 0.027	0.966	± 0.027	0.973	± 0.028	0.975	± 0.028	0.977	± 0.028	0.978	± 0.028	0.978	± 0.028	0.980	± 0.028	0.981	± 0.028	0.982	± 0.028	0.982	± 0.028
70	0.983	± 0.028	0.986	± 0.028	0.989	± 0.028	0.990	± 0.028	0.990	± 0.028	0.991	± 0.028	0.991	± 0.028	0.992	± 0.028	0.992	± 0.028	0.992	± 0.028	0.992	± 0.028
80	0.994	± 0.028	0.994	± 0.028	0.997	± 0.028	0.998	± 0.028	0.997	± 0.028	0.998	± 0.028	0.997	± 0.028	0.999	± 0.028	0.999	± 0.028	0.997	± 0.028	0.998	± 0.028
90	1.000		1.000		1.000		1.000		1.000		1.000		1.000		1.000		1.000		1.000		1.000	
100	0.996	± 0.028	0.996	± 0.028	0.997	± 0.028	0.998	± 0.028	0.997	± 0.028	0.997	± 0.028	0.997	± 0.028	0.999	± 0.028	0.998	± 0.028	0.997	± 0.028	0.996	± 0.028
110	0.984	± 0.028	0.984	± 0.028	0.987	± 0.028	0.989	± 0.028	0.989	± 0.028	0.990	± 0.028	0.991	± 0.028	0.992	± 0.028	0.992	± 0.028	0.991	± 0.028	0.992	± 0.028
120	0.965	± 0.027	0.966	± 0.027	0.971	± 0.027	0.974	± 0.028	0.975	± 0.028	0.975	± 0.028	0.977	± 0.028	0.979	± 0.028	0.980	± 0.028	0.979	± 0.028	0.980	± 0.028
130	0.936	± 0.026	0.937	± 0.027	0.944	± 0.027	0.950	± 0.027	0.954	± 0.027	0.957	± 0.027	0.959	± 0.027	0.961	± 0.027	0.963	± 0.027	0.963	± 0.027	0.965	± 0.027
140	0.895	± 0.025	0.896	± 0.025	0.909	± 0.026	0.918	± 0.026	0.924	± 0.026	0.930	± 0.026	0.934	± 0.026	0.937	± 0.027	0.940	± 0.027	0.941	± 0.027	0.945	± 0.027
150	0.836	± 0.024	0.839	± 0.024	0.859	± 0.024	0.874	± 0.025	0.885	± 0.025	0.893	± 0.025	0.900	± 0.025	0.906	± 0.026	0.910	± 0.026	0.914	± 0.026	0.919	± 0.026
160	0.748	± 0.021	0.757	± 0.021	0.788	± 0.022	0.812	± 0.023	0.830	± 0.023	0.844	± 0.024	0.855	± 0.024	0.865	± 0.024	0.872	± 0.025	0.878	± 0.025	0.883	± 0.025
170	0.627	± 0.018	0.645	± 0.018	0.696	± 0.020	0.734	± 0.021	0.762	± 0.022	0.784	± 0.022	0.801	± 0.023	0.815	± 0.023	0.826	± 0.023	0.837	± 0.024	0.847	± 0.024
180	0.542	± 0.019	0.554	± 0.019	0.600	± 0.020	0.666	± 0.021	0.701	± 0.022	0.723	± 0.022	0.753	± 0.023	0.755	± 0.023	0.775	± 0.024	0.793	± 0.024	0.800	± 0.024

## 4 DISCUSSION

In a previous study,^13^ we showed that titanium‐encapsulation of the Yb‐169 core would allow more low energy photon being transmitted through the source filter and, as a result, lead to an increased dose enhancement during GNRT, compared to stainless steel‐encapsulation. Additionally, we pointed out that the increased structural integrity of titanium over stainless steel might also provide the possibility to shrink the size of the source encapsulation, thereby further improving the dose enhancement characteristics of the source.^13^ Thus, we have focused our research effort on developing titanium‐encapsulated Yb‐169 sources, even though we also demonstrated in the aforementioned study^13^ that even stainless steel‐encapsulated Yb‐169 sources would be superior to more popular Ir‐192 sources, in terms of their dose enhancement characteristics.

As noted above, our previous publication^13^ focused on describing our research methods and findings, specifically with regards to the dose enhancement characteristics of Yb‐169 sources. As a result, there was a lack of coverage regarding more practical dosimetric issues such as the influence of titanium encapsulation on the TG‐43 parameters of Yb‐169 sources, which also need to be investigated in order to ensure the applicability of titanium‐encapsulated Yb‐169 sources to GNRT as well as conventional brachytherapy applications. Thus, this investigation was conducted to provide some insight into such issues. For example, the dose rate constant for the titanium‐encapsulated Yb‐169 source produced from this study was found comparable to the values reported for various models of stainless steel‐encapsulated Yb‐169 sources. Despite the differences in filtration due to different encapsulation designs and materials, the source anisotropy and radial dose function for the new source were also found similar to those reported for the conventional Yb‐169 sources.

## 5 CONCLUSIONS

In this study, the AAPM TG‐43 brachytherapy dosimetry parameters for a new titanium‐encapsulated Yb‐169 source were determined by MC calculations. The current results suggested that the use of titanium, instead of stainless steel, to encapsulate the Yb‐169 core would not lead to any major change in the dosimetric characteristics of the Yb‐169 source, while meeting the design goals as described in the current investigators’ published MC optimization study for GNRT applications.

## ACKNOWLEDGMENTS

This investigation was supported by the US Department of Defense, Prostate Cancer Research Program, Idea Development Award, W81XWH‐12‐1‐0198.

## CONFLICT OF INTEREST

No conflict of interest to declare.

## References

[1] Loft SM , Coles IP , Dale RG . The potential of ytterbium 169 in brachytherapy: a brief physical and radiobiological assessment. Br J Radiol. 1992;65:252–257. 1547455 10.1259/0007-1285-65-771-252

[2] Mason DL , Battista JJ , Barnett RB , Porter AT. Ytterbium‐169: calculated physical properties of a new radiation source for brachytherapy. Med Phys. 1992;19:695–703. 1508110 10.1118/1.596813

[3] Perera H , Williamson JF , Li Z , Mishra V , Meigooni AS. Dosimetric characteristics, air‐kerma strength calibration and verification of Monte Carlo simulation for a new Ytterbium‐169 brachytherapy source. Int J Radiat Oncol Biol Phys. 1994;28:953–970. 8138449 10.1016/0360-3016(94)90116-3

[4] MacPherson MS , Battista JJ . Dose distributions and dose rate constants for new ytterbium‐169 brachytherapy seeds. Med Phys. 1995;22:89–96. 7715573 10.1118/1.597597

[5] Piermattei A , Azario L , Montemaggi P . Implantation guidelines for 169 Yb seed interstitial treatments. Phys Med Biol. 1995;40:1331–1338. 7480116 10.1088/0031-9155/40/8/003

[6] Lymperopoulou G , Papagiannis P , Angelopoulos A , Karaiskos P , Georgiou E , Baltas D. A dosimetric comparison of 169Yb and 192Ir for HDR brachytherapy of the breast, accounting for the effect of finite patient dimensions and tissue inhomogeneities. Med Phys. 2006;33:4583–4589. 17278810 10.1118/1.2392408

[7] Medich DC , Tries MA , Munro JJ III . Monte Carlo characterization of an ytterbium‐169 high dose rate brachytherapy source with analysis of statistical uncertainty. Med Phys. 2006;33:163–172. 16485423 10.1118/1.2147767

[8] Lazarescu GR , Battista JJ . Analysis of the radiobiology of ytterbium‐169 and iodine‐125 permanent brachytherapy implants. Phys Med Biol. 1997;42:1727–1736. 9308079 10.1088/0031-9155/42/9/005

[9] Lymperopoulou G , Papagiannis P , Sakelliou L , Milickovic N , Giannouli S , Baltas D. A dosimetric comparison of 169 Yb versus 192 Ir for HDR prostate brachytherapy. Med Phys. 2005;32:3832–3842. 16475783 10.1118/1.2126821

[10] Das RK , Mishra V , Perera H , Meigooni AS , Williamson JF. A secondary air kerma strength standard for Yb‐169 interstitial brachytherapy sources. Phys Med Biol. 1995;40:741–756. 7652005 10.1088/0031-9155/40/5/003

[11] Piermattei A , Azario L , Rossi G , et al. Dosimetry of 169 Yb seed model X1267. Phys Med Biol. 1995;40:1317–1330. 7480115 10.1088/0031-9155/40/8/002

[12] Cazeca MJ , Medich DC , Munro JJ III . Monte Carlo characterization of a new Yb‐169 high dose rate source for brachytherapy application. Med Phys. 2010;37:1129–1136. 20384248 10.1118/1.3301607

[13] Reynoso FJ , Manohar N , Krishnan S , Cho SH. Design of an Yb‐169 source optimized for gold nanoparticle‐aided radiation therapy. Med Phys, 2014;41:101709. 25281948 10.1118/1.4895991

[14] Cho SH , Jones BL , Krishnan S . The dosimetric feasibility of gold nanoparticle‐aided radiation therapy (GNRT) via brachytherapy using low‐energy gamma‐/x‐ray sources. Phys Med Biol. 2009;54:4889–4905. 19636084 10.1088/0031-9155/54/16/004 PMC3064075

[15] Rivard MJ , Coursey BM , DeWerd LA , et al. Update of AAPM task group No. 43 report: a revised AAPM protocol for brachytherapy dose calculations. Med Phys. 2004;31:633–674. 15070264 10.1118/1.1646040

[16] Nath R , Anderson LL , Luxton G , Weaver KA , Williamson JF , Meigooni AS. Dosimetry of interstitial brachytherapy sources – recommendations of the aapm radiation‐therapy committee task group No 43. Med Phys. 1995;22:209–234. 7565352 10.1118/1.597458

[17] Medich DC , Munro JJ III . Dependence of Yb‐169 absorbed dose energy correction factors on self‐attenuation in source material and photon buildup in water. Med Phys. 2010;37:2135–2144. 20527547 10.1118/1.3372291

[18] Luxton G , Jozsef G . Radial dose distribution, dose to water and dose rate constant for monoenergetic photon point sources from 10 keV to 2 MeV:EGS4 Monte Carlo model calculation. Med Phys. 1999;26:2531–2538. 10619236 10.1118/1.598790

[19] Das RK , Meigooni AS , Misha V , Langton MA , Williamson JF. Dosimetric characteristics of the Type 8 Ytterbium‐169 interstitial brachytherapy source. J Brachytherapy Int. 1997;13:219–234.

[20] Mainegra E , Capote R , Lopez E . Dose rate constants for I‐125, Pd‐103, Ir‐192 and Yb‐169 brachytherapy sources: an EGS4 Monte Carlo study. Phys Med Biol. 1998;43:1557–1566. 9651025 10.1088/0031-9155/43/6/014

